# Long noncoding RNA TUG1 induces angiogenesis of endothelial progenitor cells and dissolution of deep vein thrombosis

**DOI:** 10.1186/s12959-022-00413-y

**Published:** 2022-09-26

**Authors:** Yaping Feng, Bo Lei, Huan Zhang, Luyuan Niu, Xiangtao Li, Xiaoyun Luo, Fuxian Zhang

**Affiliations:** 1 Department of Vascular Surgery, Beijing Shijitan Hospital, Capital Medical University, Beijing, 100038 China; 2 Anesthesia Department, Beijing Haidian Maternal & Child Health Hospital, No. 33 Haidian South Road, Haidian District, Beijing, 100080 China

**Keywords:** Deep vein thrombosis, Endothelial progenitor cells, Long noncoding RNA taurine upregulated gene 1, microRNA-92a-3p, 3-Hydroxy-3-methylglutaryl coenzyme a reductase, Angiogenesis

## Abstract

**Objective:**

Long non-coding RNA (lncRNA) essentially controls many physiological and pathological processes of deep vein thrombosis (DVT). Based on that, lncRNA taurine upregulated gene 1 (TUG1)-involved angiogenesis of endothelial progenitor cells (EPCs) and dissolution of DVT was explored.

**Methods:**

In the in-vitro experiments, EPCs were engineered with mimic, inhibitor, siRNA, and plasmid, after which tube formation, proliferation, migration, and apoptosis were checked. In the in-vivo experiments, a DVT mouse model was established. Before the DVT operation, the mice were injected with agomir, antagomir, siRNA, and plasmid. Subsequently, thrombosis and damage to the femoral vein were pathologically evaluated. TUG1, miR-92a-3p, and 3-Hydroxy-3-methylglutaryl coenzyme A reductase (Hmgcr) expression in the femoral vein was tested. The relationship between TUG1, miR-92a-3p, and Hmgcr was validated.

**Results:**

DVT mice showed suppressed TUG1 and Hmgcr expression, and elevated miR-92a-3p expression. In EPCs, TUG1 overexpression or miR-92a-3p inhibition promoted cellular angiogenesis, whereas Hmgcr silencing blocked cellular angiogenesis. In DVT mice, elevated TUG1 or inhibited miR-92a-3p suppressed thrombosis and damage to the femoral vein whilst Hmgcr knockdown acted oppositely. In both cellular and animal models, TUG1 overexpression-induced effects could be mitigated by miR-92a-3p up-regulation. Mechanically, TUG1 interacted with miR-92a-3p to regulate Hmgcr expression.

**Conclusion:**

Evidently, TUG1 promotes the angiogenesis of EPCs and dissolution of DVT via the interplay with miR-92a-3p and Hmgcr.

**Supplementary Information:**

The online version contains supplementary material available at 10.1186/s12959-022-00413-y.

## Introduction

Deep vein thrombosis (DVT) is a common manifestation of venous thromboembolism (VTE) and is defined as a form of thrombophlebitis associated with the formation of blood clots in deep veins. DVT is sometimes asymptomatic but often presents as non-specific symptoms, such as discomfort or pain in the legs, or a feeling of fever. Pain, tenderness, swelling or blue or red discoloration of the limbs are the typical symptoms [1]. Oral anticoagulants are the first-line treatment for VTE to hasten thrombus resolution because of lower bleeding risk. It takes at least 3 months to take anticoagulants to prevent early recurrence [2]. Endothelial progenitor cells (EPCs) could modify endothelial regeneration, revascularization, vascular activity and angiogenic factor secretion, protease production, thrombosis and recurrence prevention, and vein wall remodeling [3]. In addition, EPCs can form new blood vessels by differentiating into endothelial cells, which could be utilized as a promising therapeutic regimen for DVT-associated thrombus resolution in patients achieved limited success [4]. Therefore, targeting EPCs is a promising direction for the resolution of DVT.

Several long noncoding RNAs (lncRNAs) are listed as essential partners in multitudinous physiological and pathological processes of DVT, including proliferation, migration, and angiogenesis of EPCs [4, 5]. Specifically, lncRNA taurine upregulated gene 1 (TUG1) is a unique modifier for EPC function and angiogenesis under diabetic conditions [6]. Moreover, a report on aneurysms proposes that TUG1 overexpression induces EPC migration, invasion, and differentiation [7]. But, little is known about TUG1-mediated influence on EPCs angiogenesis in the setting of DVT. According to the latest annotation by the Ensembl database, there are 8 transcripts (isoforms) of Tug1 (https://www.ensembl.org/Mus_musculus/Gene/Summary?db=core;g=ENSMUSG00000056579;r=11:3589785-3599673). Tug1–202 is a transcript with length ≥ 200 bp and number of exons ≥2. After calculation, the minimum coverage of TUg1–202reads is ≥3, so subtype Tug1–202 was selected for our study. LncRNAs-mediated competitive binding of microRNAs (miRs) plays a vital role in regulating RNA transcription. It has been widely explored that various miRs involve in the pathological possesses of DVT, including vascular endothelial cell physiology [8], autophagy and tube formation of EPCs [9], and recanalization and resolution [10]. It has been implicated that miR-92 could modify vascular smooth muscle cell function in vascular restenosis and injury [11]. miR-92a-3p is associated with oxidative stress in central venous catheter-related thrombosis (CRT) [12], and miR-92a-3p inhibition could hasten angiogenesis of endothelial cells, serving as a potential target for the treatment of atherosclerosis [13]. 3-Hydroxy-3-methylglutaryl coenzyme A reductase (Hmgcr) is the rate-limiting enzyme for the biosynthesis of cholesterol and isoprenoids. Jayoung Choi et al. have specified that mevalonate, a metabolic product of Hmgcr, could restore venous angiogenesis [14].

Based on former publications, we studied TUG1-mediated EPCs angiogenesis and DVT resolution via miR-92a-3p/Hmgcr axis, expecting to discover a molecular way for the disease management.

## Materials and methods

### Ethical approval

This research was processed with the approval of the animal ethic committee of Beijing Haidian Maternal & Child Health Hospital.

### Isolation and culture of EPCs

Using Histopaque 1077 density gradient centrifugation (Sigma-Aldrich, MO, USA), bone marrow mononuclear cells (MNCs) were isolated from C57 mice. MNCs/cm^2^ (10^6^) were seeded on fibronectin-coated six-well plates in endothelial cell growth medium-2MV (EGM-2MV, Lonza, MD, USA) containing 10% fetal bovine serum, 1% streptomycin and penicillin, and then cultivated in a constant temperature incubator at 37 °C with 5% CO_2_. The adherent cells were removed 72 h later. Since that, the medium was renewed every 3 days [15].

After 10 d, adherent cells were fixed with 2.5 mg/mL DiI-acetylated-low density lipoprotein (DiI-ac-LDL, Peking Union-Biology, Beijing, China) for 2 h and with 2% paraformaldehyde (Sigma) for 5 min. Subsequently, cells were incubated with 10 mg/L fluorescein isothiocyanate labeled ulex europaeus agglutinin (Sigma) for 1 h.

EPCs were incubated with primary antibodies CD133 and FLK-1 (both from Abcam, Cambridge, UK) and combined with Cy3 (BOSTER, Wuhan, China) or fluorescein isothiocyanate (FITC; Santa Cruz, CA, USA). Representative micrographs were obtained with a microscope (Olympus, Tokyo, Japan) [16].

### EPCs transfection

Lipofectamine 3000 (Invitrogen, CA, USA) was utilized for EPCs transfection. The transfection plans included oe-NC, oe-TUG1, NC inhibitor, miR-92a-3p inhibitor, NC siRNA, Hmgcr siRNA, oe-TUG1 + NC mimic, oe-TUG1 + miR-92a-3p mimic, miR-92a-3p inhibitor + NC siRNA and miR-92a-3p inhibitor + Hmgcr siRNA (Antpedia, Shanghai, China). Cells were collected after 48 h of transfection.

### In vitro tube formation experiment

Growth factor-reduced Matrigel (BD Biosciences, NJ, USA) was solidified in 96-well plates at 50 μL/well. EPCs (1 × 10^4^) were resuspended in 200 μL endothelial basal medium-2 without EGM-2 SingleQuots, and added onto Matrigel. Tube images were captured after 18 h, and the number of tubes was recorded [17].

### Cell counting kit (CCK)-8 test

EPCs pre-cultured in 96-well plates at 5000 cells/well were added with CCK-8 solution (Dojindo, Kumamoto, Japan) at 0, 24, 48, and 72 h, respectively. Absorbance was measured at 450 nm with a microplate reader (BioTek Instruments, VT, USA).

### Flow cytometry

Detection of apoptosis was carried out following the instruction of FITC Annexin V/propidium iodide Apoptosis Detection Kit I (Ribobio, Guangzhou, China). Apoptosis of EPCs was examined on a FACScan flow cytometer (Becton Dickinson, NJ, USA), and data analysis was done by FlowJo software. The upper left quadrant (Q1) was necrotic cells: negative for FITC Annexin V staining and positive for PI staining; the upper right quadrant (Q2) was late apoptotic cells: positive for FITC Annexin V staining and positive for PI staining; the lower right quadrant (Q3) was early apoptotic cells: positive for FITC Annexin V staining and negative for PI staining; the lower left quadrant (Q4) was live cells: negative for FITC Annexin V staining and negative for PI staining [18].

### Transwell detection

EPCs (1 × 10^5^) were placed in the top compartment pretreated with Matrigel whereas EBM-2MV supplemented with 20% fetal bovine serum was in the lower compartment. The membrane was stained with 0.1% crystal violet after 24-h cell activity, and the number of EPCs permeating the membrane was calculated under an optical microscope (Olympus) [19].

### Establishment of DVT animal models

Thirty minutes before DVT operation, various constructs at 10 nmol (oe-NC, oe-TUG1, NC antagomir, miR-92a-3p antagomir, NC siRNA, Hmgcr siRNA, oe-TUG1 + NC agomir, oe-TUG1 + miR-92a-3p agomir, miR-92a-3p antagomir + NC siRNA, and miR-92a-3p antagomir + Hmgcr siRNA) were dissolved in 200 μL normal saline and injected into mice through the tail vein. A DVT mouse model was established according to the method previously reported [20]. In the sham group, occlusion of the femoral veins on both sides was not performed. The mice were euthanized 24 h after the operation, and a fresh thrombus was taken and weighed [21, 22].

### Observation of thrombus

Fresh thrombus was sliced, observed under an optical microscope (XP-330, Shanghai Bingyu Optical Instrument Co., Ltd., Shanghai, China), and pathologically evaluated. 0 point meant no thrombosis, 1 point meant vascular occlusion < 50%, 2 points meant vascular occlusion > 50% (incomplete occlusion), and 3 points meant complete vascular occlusion [23].

### Hematoxylin-eosin (HE) staining

Femoral vein samples of mice were taken, and a 1 cm blood vessel was cut from each vein and fixed with 10% neutral formaldehyde. The blood vessels were paraffin-embedded and cut into 4 μm sections for hematoxylin and 1% eosin staining. After that, images were obtained under an optical microscope (OLYMPUS, Tokyo, Japan) and data analysis was performed by Image-Pro Plus 6.0 software (IPP6.0, Media Cybernetics, MD, USA) [24].

### Quantitative PCR analysis

Trizol (Thermo Fisher Scientific) was adopted to extract the total RNA from cells and tissues. Before reverse transcription, RNase-free DNase I was used to remove possible DNA contamination such as genomic DNA in the extracted total RNA. PrimeScript RT reagent kit (Takara) or microRNA reverse transcription system (GenePharma, Shanghai, China) was implemented to synthesize cDNA. SYBR Green quantitative PCR Master Mix (Takara) or miRNAs Quantitation Kit (GenePharma) was used for RT-qPCR analysis. miR-92a-3p expression was standardized by U6, while other genes were standardized by GAPDH. The 2^^-ΔΔCT^ method was used to calculate the relative expression of each gene. RT-qPCR primers were supplemented in Supplementary Table 1 [4].

### Immunoblotting analysis

Total protein in tissue and cells was extracted with radio-immunoprecipitation assay lysis buffer, followed by sodium dodecyl sulphate polyacrylamide gel electrophoresis. Then, a 5% skimmed milk-blocked polyvinylidene fluoride protein membrane (Micropore, Sigma) was incubated with primary antibody against GAPDH (1:1000, Abcam) and Hmgcr (1:1000, Abcam), and with the appropriate secondary antibody. After shooting with the image analysis system (Bio-Rad), the immune complex was visualized using an enhanced chemiluminescence kit (Amsham, UK) [25].

### RNA immunoprecipitation (RIP) assay

EPC lysate collected by RIP lysis buffer (protease inhibitor and RNase inhibitor) was cultivated with protein-G/A plus agarose and Ago2 antibody (Abcam) or immunoglobulin G (IgG; Abcam). The precipitated RNA was extracted using Trizol (Thermo Fisher Scientific) following the manufacturer’s protocol and was subjected to RT-qPCR analysis [26].

### Luciferase reporter assay

Amplified wild type (Wt)-TUG1/mutant (Mut)-TUG1 or Wt-Hmgcr 3’UTR/Mut-Hmgcr 3’UTR sequence was cloned into PGL3 basic vector (Promega, WI, USA). The reporter was co-transfected with NC mimic and miR-92a-3p mimic into EPCs. Luciferase activity was measured using a dual luciferase reporter gene assay system (Promega) after 48 h [27].

### Chromatin immunoprecipitation (ChIP) assay

ChIP was performed via Pierce Sepharose Chip Kit (Thermo Fisher Scientific). Cells were cross-linked in 1% formaldehyde and quenched by glycine solution. The cell pellets were lysed with micrococcal nuclease, and the supernatant was reacted with anti-Hmgcr (1:200, Abcam) or IgG, and precipitated with Protein A/G Sepharose beads (Thermo Fisher Scientific). The protein/DNA complex was eluted by proteinase K, and genomic DNA fragments were analyzed by quantitative PCR.

### Statistics

Data were analyzed using SPSS 21.0 (IBM, NY, USA), and the measurement data were represented by mean ± standard deviation. The normal distribution and variance homogeneity were initially measured. As for data fitting normal distribution and variance homogeneity, an unpaired two-tailed Student’s t-test was utilized when comparing two experimental groups, while three experimental groups were analyzed using one-way analysis of variance (ANOVA) followed by Tukey’s post hoc test. *P* < 0.05 was meaningful for statistical significance.

## Results

### EPCs identification and DVT mouse model establishment

**Fig. 1:**
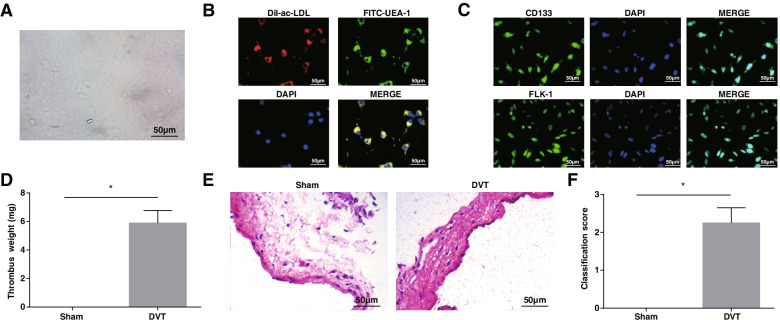
EPCs identification and DVT mouse model construction. **A** EPCs were cultured for 7 days and showed a cobblestone-like morphology; **B** EPCs uptake of FITC-UEA combined with DiI-ac-LDL; **C** Immunofluorescence detection of CD133 and FLK-1; **D** Weight of mouse thrombus; **E** HE staining of femoral vein of mice; **F** Thrombosis score of mice; * *P* < 0.05. In vitro experiments were undertaken on 3 biological and 3 technical replicates, and in vivo experiments were undertaken on *n* = 5 animals per group

The isolated cells showed a cobblestone-like morphology (Fig. 1A), and could be combined with DiI-ac-LDL and FITC-UEA (Fig. 1B), indicating the differentiation of EPCs. Immunofluorescence staining confirmed the cells as EPCs based on the expression of the cellular surface antigens CD133 and FLK-1, which were standard EPC markers (Fig. 1C).

After the DVT operation, the mice were euthanized and the thrombus was weighed. It was found that the thrombus was heavier in mice after DVT operation (Fig. 1D). HE staining exhibited that normal mouse femoral vein tissue structure was intact and undamaged, spindle-shaped endothelial cells were uniformly arranged and uniform in size; vascular smooth muscle cells were neatly arranged, and the intima was smooth and even. After the DVT operation, disorders of the endothelium, vascular endothelial cells, vascular smooth muscle, a large amount of focal inflammation, and exudation of the vessel wall and interstitial tissue were seen, the vessel wall became thinner, and the thrombus extended to the vessel lumen (Fig. 1E). Meanwhile, increased thrombus score was observable in DVT mice (Fig. 1F). In summary, EPCs were successfully isolated and the mouse DVT model was successfully established.

### TUG1 promotes angiogenesis of EPCs and resolution of DVT

**Fig. 2:**
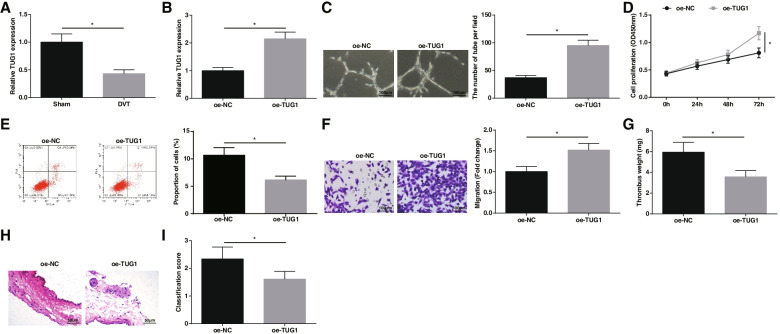
TUG1 promotes angiogenesis of EPCs and resolution of DVT. **A** TUG1 expression in mice after DVT operation; **B** TUG1 expression in EPCs; **C** Tube formation of EPCs; **D** EPCs proliferation; **E** EPCs apoptosis; **F** EPCs migration; **G** Weight of mouse thrombus; **H** HE staining of femoral vein of mice; **I** Thrombosis score of mice; # *P* < 0.05 vs. the sham group; * *P* < 0.05. In vitro experiments were undertaken on 3 biological and 3 technical replicates, and in vivo experiments were undertaken on *n* = 5 animals per group

TUG1 expression was checked by RT-qPCR, showing that TUG1 expression in DVT mice was low (Fig. 2A). Oe-TUG1 construct was transfected into EPCs, and RT-qPCR confirmed that TUG1 was successfully up-regulated (Fig. 2B). In vitro angiogenesis, CCK-8, flow cytometry, and Transwell experiments indicated that TUG1-overexpressed EPCs had a stronger ability of angiogenesis, proliferation, and migration, accompanied by reduced apoptosis rate (Fig. 2C-F).

In the mouse model of DVT, the oe-TUG1 injection was performed. It was addressed that TUG1 overexpression decreased the weight of thrombus (Fig. 2G), attenuated vascular cell disorder and inflammation, thickened blood vessel wall, reduced the area of thrombus (Fig. 2H), and reduced the thrombosis score of DVT mice (Fig. 2I). Taken together, TUG1 can promote EPCs angiogenesis and DVT resolution.

### Interaction between TUG1 and miR-92a-3p

**Fig. 3:**
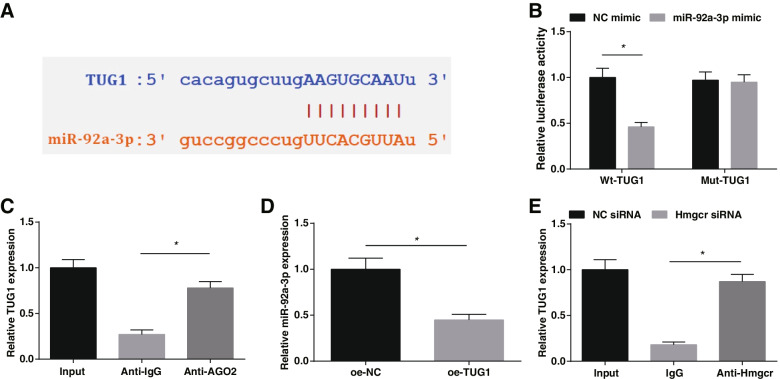
Interaction between TUG1 and miR-92a-3p. **A** The existence of binding sites of TUG1 and miR-92a-3p; **B** Effect of miR-92a-3p on luciferase activity of Wt-TUG1 and Mut-TUG1 reporters; **C** TUG1 enrichment under Ago2; **D** Effect of TUG1 on miR-92a-3p expression in EPCs; **E** ChIP experiment; * *P* < 0.05. In vitro experiments were undertaken on 3 biological and 3 technical replicates, and in vivo experiments were undertaken on *n* = 5 animals per group

The binding sites of TUG1 and miR-92a-3p were predicted by Starbase (http://starbase.sysu.edu.cn/) (Fig. 3A). Luciferase activity assay and RIP experiment further verified the binding relationship between TUG1 and miR-92a-3p, as the results shown that the luciferase activity of EPCs transfected with Wt-TUG1 and miR-92a-3p mimic decreased (Fig. 3B); AGO2 had a promoting effect on the enrichment of TUG1 (Fig. 3C). RT-qPCR suggested that when TUG1 expression increased, miR-92a-3p expression was decreased (Fig. 3D). ChIP experiment disclosed the enrichment of Hmgcr on the promoter of TUG1 (Fig. 3E). The above results indicate that TUG1 has a modification impact on miR-92a-3p expression.

### Down-regulating miR-92a-3p promotes angiogenesis and resolution of DVT

**Fig. 4:**
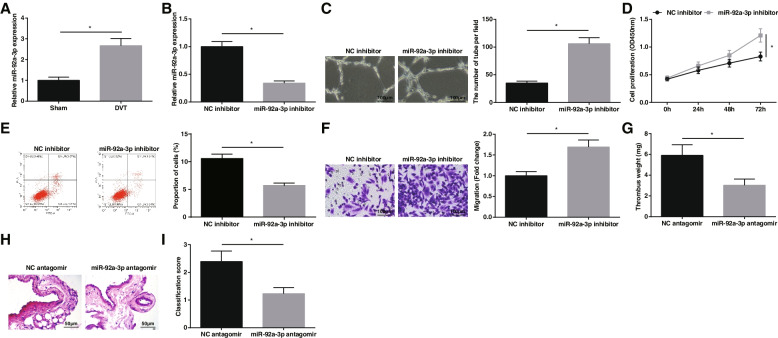
Down-regulating miR-92a-3p promotes resolution of DVT. **A** miR-92a-3p expression in mice after DVT operation; **B** miR-92a-3p expression in EPCs; **C** Tube formation of EPCs; **D** EPCs proliferation; **E** EPCs apoptosis; **F** EPCs migration; **G** Weight of mouse thrombus; **H** HE staining of femoral vein of mice; **I** Thrombosis score of mice; * *P* < 0.05. In vitro experiments were undertaken on 3 biological and 3 technical replicates, and in vivo experiments were undertaken on *n* = 5 animals per group

As reported, miR-92a-3p is up-regulated in CRT [12]. In a mouse-based DVT model, RT-qPCR suggested that miR-92a-3p was highly expressed (Fig. 4A). Aiming to decipher the miR-92a-3p-related mechanism in EPCs angiogenesis, miR-92a-3p expression in EPCs was modified by transfection with miR-92a-3p inhibitor (Fig. 4B). Subsequently, cellular experiments presented that miR-92a-3p-depleted EPCs had promoted tube formation, proliferation, and migration abilities, and decreased apoptosis rate (Fig. 4C-F).

miR-92a-3p expression in DVT mice was downregulated by tail vein injection with miR-92a-3p antagomir. It was found that miR-92a-3p suppression could reduce the weight of thrombus (Fig. 4G), relieved thrombosis (Fig. 4H), and reduced the thrombosis score of DVT mice (Fig. 4I). All in all, down-regulating miR-92a-3p induces EPCs angiogenesis and resolution of DVT.

### Target relation between miR-92a-3p and Hmgcr

**Fig. 5:**
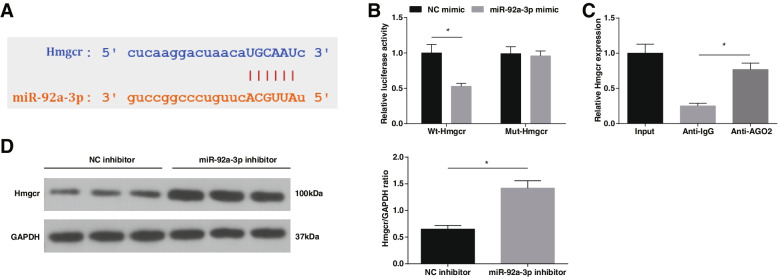
Target relation between miR-92a-3p and Hmgcr. **A** The existence of binding sites of miR-92a-3p and Hmgcr; **B** Effect of miR-92a-3p on luciferase of Wt-Hmgcr and Mut-Hmgcr reporters; **C** Hmgcr enrichment under Ago2; **D** Effect of miR-92a-3p on Hmgcr protein expression in EPCs; * *P* < 0.05. In vitro experiments were undertaken on 3 biological and 3 technical replicates, and in vivo experiments were undertaken on *n* = 5 animals per group

Binding sites existed between miR-92a-3p and Hmgcr according to the result of Starbase (Fig. 5A). Luciferase reporter test presented that the luciferase activity of EPCs co-transfected with miR-92a-3p mimic and Wt-Hmgcr was significantly decreased, while the luciferase activity of EPCs transfected with Mut-Hmgcr and miR-92a-3p mimic did not change significantly (Fig. 5B); RIP test examined that Hmgcr enrichment was seen under AGO2 condition (Fig. 5C). Western blot showed that Hmgcr expression was promoted in the presence of miR-92a-3p down-regulation (Fig. 5D). Precisely, a negative interaction exists between miR-92a-3p and Hmgcr.

### Knockdown of Hmgcr inhibits EPCs angiogenesis and aggravated thrombosis formation

**Fig. 6:**
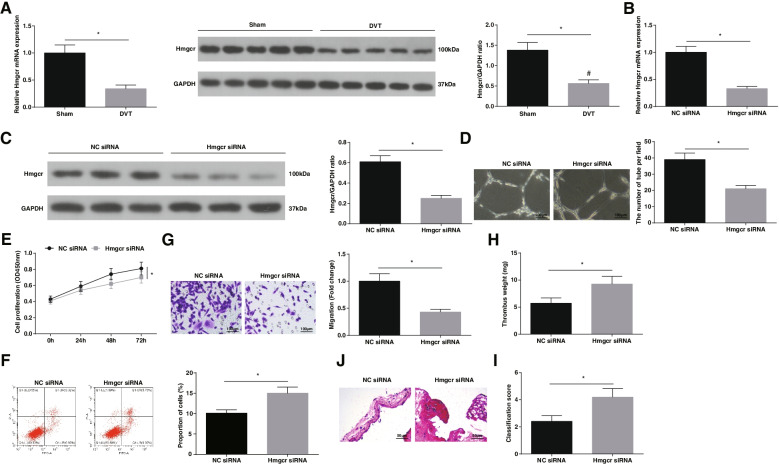
Knockdown of Hmgcr aggravates thrombosis formation. **A** Hmgcr mRNA and protein expression in mice after DVT operation; **B**-**C** Hmgcr mRNA and protein expression in EPCs; **D** Tube formation of EPCs; **E** EPCs proliferation; **F** EPCs apoptosis; **G** EPCs migration; **H** Weight of mouse thrombus; **I** HE staining of femoral vein of mice; **J** Thrombosis score of mice; * *P* < 0.05. In vitro experiments were undertaken on 3 biological and 3 technical replicates, and in vivo experiments were undertaken on *n* = 5 animals per group

It has been discussed that injection of mevalonate, a metabolite of Hmgcr, can induce venous angiogenesis [14]. RT-qPCR and Western blot demonstrated that Hmgcr expression was deficient in mice with DVT (Fig. 6A). For clarifying the Hmgcr-mediated process of EPCs angiogenesis, Hmgcr expression was regulated in EPCs by Hmgcr siRNA (Fig. 6B, C). Based on Hmgcr silencing, fewer tubes, decreased proliferation and migration, in concert with increased apoptosis rate of EPCs were detectable (Fig. 6D-G).

Hmgcr siRNA injection was implemented in DVT mice, causing thrombus weight increase (Fig. 6H), thrombus aggravate (Fig. 6I), and thrombosis score elevate (Fig. 6J). In conclusion, knocking down Hmgcr inhibits EPCs angiogenesis and DVT resolution.

### The interplay of TUG1/miR-92a-3p/Hmgcr in the resolution of DVT

**Fig. 7:**
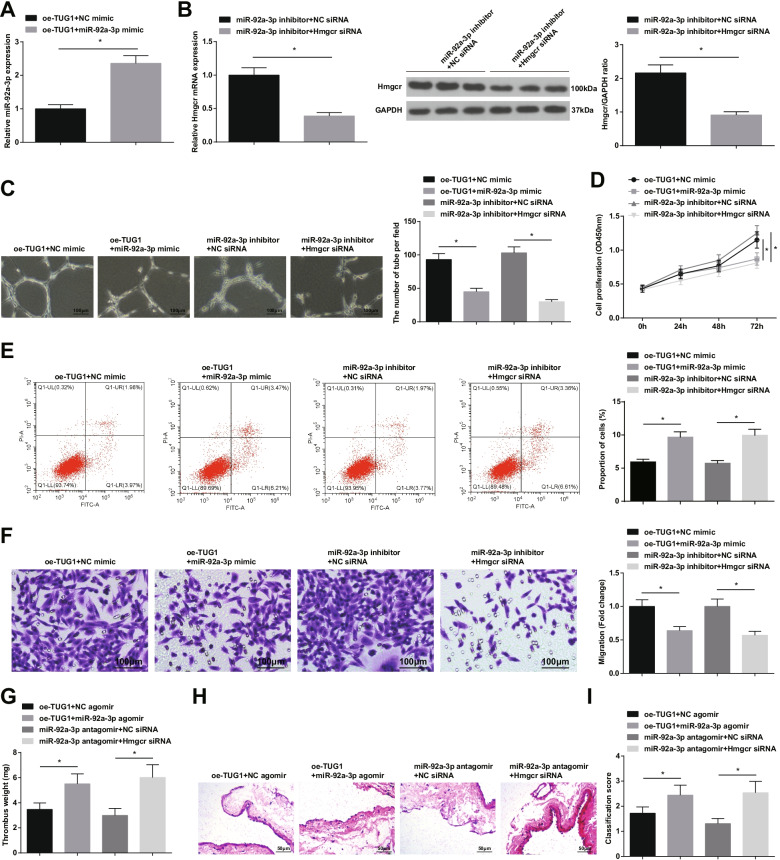
The interplay of TUG1/miR-92a-3p/Hmgcr in the resolution of DVT. **A** miR-92a-3p expression in EPCs; **B** Hmgcr mRNA and protein expression in EPCs; **C** Tube formation of EPCs; **D** EPCs proliferation; **E** EPCs apoptosis; **F** EPCs migration; **G** Weight of mouse thrombus; **H** H&E staining of femoral vein of mice; **I** Thrombosis score of mice; * *P* < 0.05. In vitro experiments were undertaken on 3 biological and 3 technical replicates, and in vivo experiments were undertaken on *n* = 5 animals per group

In in vitro EPCs, miR-92a-3p mimic was transfected based on oe-TUG1 interference (Fig. 7A); meanwhile, Hmgcr siRNA was introduced into EPCs based on miR-92a-3p inhibitor treatment (Fig. 7B). Data obtained from cell-based tests revealed that EPCs angiogenesis induction mediated by oe-TUG1 or miR-92a-3p inhibitor could be counteracted by miR-92a-3p mimic or Hmgcr siRNA, respectively (Fig. 7C-F).

Animal experiments found that miR-92a-3p agomir or Hmgcr siRNA could respectively rescue the effects of oe-TUG1 or miR-92a-3p antagomir on the weight of thrombus (Fig. 7G), femoral vein pathological damage (Fig. 7H), and thrombosis score (Fig. 7I) of DVT mice.

## Discussion

VTE is the third most frequent cardiovascular disease besides myocardial infarction and stroke [28]. In a cell model and a mouse model, we confirmed that TUG1 overexpression promoted angiogenesis of EPCs and resolution of DVT in mice via miR-92a-3p down-regulation and subsequent Hmgcr up-regulation.

TUG1 is involved in a variety of cell signaling pathways and tissue-specific expressions. Its size is 7.1 kb, which is large enough to fold into a complex secondary/tertiary structure [29]. It has been indicated that TUG1 expression is constrained in acute lung injury based on a mouse model, and TUG1 overexpression could protect primary murine pulmonary microvascular endothelial cells from lipopolysaccharide (LPS)-induced apoptosis [30]. For EPCs, TUG1 restoration rescues high glucose-induced decline of cellular migration, invasion, and tube formation abilities, while for diabetic mice, TUG1 overexpression induces angiogenesis in the ischemic limbs [6]. In a cell model of sepsis, TUG1 up-regulation could present human umbilical vein endothelial cells from LPS-induced apoptosis [31]. As indicated previously, TUG1 induction is multifunctional regarding proliferation, invasion, and angiogenesis promotion of trophoblasts in the setting of preeclampsia [32]. Long J et al. have supported that podocyte-specific elevation of TUG1 could improve diabetic nephropathy-associated biochemical and histological features in mice [33]. In our work, examination of TUG1 expression indicated that TUG1 was poorly expressed in DVT mice. Concerning the action of TUG1, our experimental observations presented that restoration of TUG1 accelerated proliferation, migration, and tube-forming abilities whilst decelerated apoptosis of EPCs; in DVT mice, overexpressed TUG1 exerted to decrease thrombus, and relieve femoral vein pathological damage.

Many articles have demonstrated that TUG1 is a sponge for many miRNAs [34–37]. In the preliminary research, we found that miR-92a-3p was targeted by TUG1 by the bioinformatics website Starbase (https://starbase.sysu.edu.cn/agoClipRNA.php). Therefore, we chose miR-92a-3p as the research direction. miR-92a-3p is a quantified miRs involved in the regulation of vascular performance, and it is up-regulated in patients with coronary artery disease [38], as well as in high-density lipoprotein fraction of early diabetic mice after ischemia [39]. It has been elaborated that miR-92a-3p is associated with endothelial dysfunction, and aberrant elevation of miR-92a-3p expression is recorded in the venous tissue of rats with CRT [12]. On the other hand, endothelial miR-92a-3p expression is induced after renal injury, and dual suppression of miR-92a-3p/miR-489-3p can relieve atherosclerosis based on a mouse model [40]. It is well-established that up-regulated miR-92a is involved in endothelial injury, and suppression of miR-92a is feasible to protect endothelial cells in response to acute myocardial infarction [41]. It is known that hypoxia or high glucose induces injury of EPCs, in which miR-92a suppression could enhance cellular migration and tube formation abilities [42]. To improve neovascularization, Shyu KG et al. have verified that hyperbaric oxygen has valuable therapeutic effects partly through enhancing lncRNA metastasis-associated lung adenocarcinoma transcript 1-mediated down-regulation of miR-92a [43]. Based on the sponge adsorption phenomenon between TUG1 and miR-92a-3p, the role of miR-92a-3p was surveyed in detail. The findings displayed that miR-92a-3p inhibition similarly phenocopied the impacts of overexpressed TUG1 on EPCs in vitro and DVT mice in vivo.

Angiogenesis is regulated by the Hmgcr pathway through the differentially regulated arteriovenous demand for protein prenylation in endothelial cells [14]. Hmgcr function impairment disturbs the stability of cerebral blood vessels, leading to the progressive expansion of blood vessels and subsequent rupture of blood vessels [44]. From our analysis, it was noticeable that miR-92a-3p had a targeted regulatory effect on Hmgcr expression. Given that, we subsequently found that Hmgcr suppression blocked EPCs’ activities, and aggravated DVT in mice.

All in all, our study analysis exhibited the TUG1-induced protection against DVT through interacting with miR-92a-3p and Hmgcr. This research has delved into a brand-new way to treat DVT from the molecular TUG1/miR-92a-3p/Hmgcr cascade. One of the study limitations is that the signaling pathway involved in the TUG1/miR-92a-3p/Hmgcr axis-regulated DVT resolution was not explored. Additionally, TUG1 is a sponge of the many miRNAs, and miR-92a-3p is not the only miRNA responsible for the phenotype observed in this study, therefore, further research is warranted to validate our findings.

## Supplementary Information

**Additional file 1: Supplementary Table 1.** Sequences for qPCR.

## Data Availability

The original contributions presented in the study are included in the article/Supplementary Material, further inquiries can be directed to the corresponding author.
