# Supplementary material for: Long noncoding RNA TUG1 induces angiogenesis of endothelial progenitor cells and dissolution of deep vein thrombosis
Source: Thromb J. 2022 Sep 26;20:54. doi: 10.1186/s12959-022-00413-y (PMC9511754; doi:10.1186/s12959-022-00413-y)
Supplement: Supplementary file 1 — Additional file 1: Supplementary Table 1. Sequences for qPCR. [file 12959_2022_413_MOESM1_ESM.docx]

**Supplementary Table 1** Sequences for qPCR

| Genes | Primer sequences (5’-3’) |
| --- | --- |
| TUG1 | F: CAAGAAACAGCAACACCAGAAG |
|  | R: TAAGGTCCCCATTCAAGTCAGT |
| Hmgcr | F: ATCCTGACGATAACGCGGTG |
|  | R: AAGAGGCCAGCAATACCCAG |
| GAPDH | F: GGTGAAGGTCGGTGTGAACG |
|  | R: TGTAGACCATGTAGTTGAGGTCA |

Note: F, forward; R, reverse; miR-92a-3p, microRNA-92a-3p; TUG1, long noncoding RNA taurine upregulated gene 1; Hmgcr, 3-Hydroxy-3-methylglutaryl coenzyme A reductase; GAPDH, glyceraldehyde-3-phosphate dehydrogenase
